# A Val292 substitution combined with an alanine duplication (ADUP) in the Ω loop of ADC β-lactamase confers reduced susceptibility to advanced β-lactam agents, including cefiderocol

**DOI:** 10.1128/mbio.03518-25

**Published:** 2026-04-08

**Authors:** Akito Kawai, Christi L. McElheny, Ryan K. Shields, Cesar A. Arias, David L. Paterson, Robin Patel, Robert A. Bonomo, Vance G. Fowler, David van Duin, Yohei Doi

**Affiliations:** 1 Department of Microbiology, Fujita Health University School of Medicine 89305, Toyoake, Japan; 2 Center for Innovative Antimicrobial Therapy, Division of Infectious Diseases, University of Pittsburgh School of Medicine, Pittsburgh, Pennsylvania, USA; 3 Division of Infectious Diseases, Houston Methodist Hospital 23534 , Houston, Texas, USA; 4 Center for Infectious Diseases, Houston Methodist Research Institute 167626 , Houston, Texas, USA; 5 Department of Medicine, Weill Cornell Medical College 12295 , New York, New York, USA; 6 ADVANCE-ID, Saw Swee Hock School of Public Health, National University of Singapore 37580, Singapore, Singapore; 7 Department of Medicine, National University of Singapore 37580, Singapore, Singapore; 8 Division of Clinical Microbiology, Department of Laboratory Medicine and Pathology and Division of Infectious Diseases, Department of Medicine, Mayo Clinic, Rochester, Minnesota, USA; 9 Case Western Reserve University University-Veteran Affairs Center for Antimicrobial Resistance and Epidemiology (Case VA CARES), Research and Medical Services, Louis Stokes Cleveland Department of Veterans Affairs Medical Center 2546, Cleveland, Ohio, USA; 10 Department of Medicine, Pharmacology, Molecular Biology and Microbiology, and Biochemistry, Case Western Reserve University School of Medicine 12304, Cleveland, Ohio, USA; 11 Duke Clinical Research Institute, Duke University, Durham, North Carolina, USA; 12 Division of Infectious Diseases, University of North Carolina, Chapel Hill, North Carolina, USA; 13 Department of Infectious Diseases, Fujita Health University School of Medicine 89305, Toyoake, Japan; University of Texas at Dallas, Richardson, Texas, USA

**Keywords:** cefiderocol, beta-lactamase, ADC-227, ADC-33 variant, carbapenem-resistant *Acinetobacter baumannii*

## Abstract

Intrinsic *Acinetobacter*-derived cephalosporinases (ADCs) in *Acinetobacter baumannii* are AmpC-type β-lactamases that confer resistance to β-lactam agents, typically through insertion sequence (IS) element-driven overexpression. However, the contribution of ADCs to resistance against advanced β-lactam agents has not been systematically investigated. Given the increasing clinical use of these agents, including cefiderocol, we analyzed the diversity and function of ADC variants in a global collection of carbapenem-resistant *A. baumannii* (CRAb) isolates. We identified 52 distinct ADC variants in a collection of 428 CRAb clinical isolates from the United States. Among these, variants carrying both a valine substitution at position 292 in the R2 loop and an alanine duplication (ADUP) in the Ω loop consistently conferred stable resistance to cefiderocol. Biochemical and crystallographic analyses demonstrated that ADC-227, which harbors a V292W substitution with an ADUP at position 218a in the Ω loop, exhibits enhanced catalytic efficiencies for ceftazidime and cefiderocol and moderately reduced inhibition by avibactam, leading to resistance not only to cefiderocol but also to ceftazidime–avibactam. Structural studies revealed conformational flexibility of the R2 loop, allowing dynamic accommodation of substrates. Collectively, the findings identify Val292, in combination with an ADUP in the Ω loop, as a mutational “hot spot” for ADCs evolution that may undermine the efficacy of newer β-lactams, including cefiderocol. These results underscore the need for ongoing molecular surveillance of *A. baumannii* isolates to detect and track the emergence of such variants in clinical settings.

**IMPORTANCE:**

Carbapenem-resistant *Acinetobacter baumannii* (CRAb) has been designated as a critical priority pathogen by the World Health Organization (WHO). Cefiderocol has been introduced as a novel therapy against CRAb; however, recent clinical data highlight concerning treatment failures and excess mortality. Understanding resistance mechanisms is therefore essential to preserve the clinical utility of this agent. This study addresses a critical knowledge gap by investigating the role of intrinsic *Acinetobacter*-derived cephalosporinases (ADCs), which are ubiquitous in *A. baumannii* and diverse in sequence. By defining specific mutational patterns that endanger cefiderocol activity, this work highlights how chromosomally encoded enzymes can evolve to erode the effectiveness of newer β-lactams such as cefiderocol. These insights underscore the importance of integrating molecular surveillance into clinical practice and antimicrobial stewardship to ensure timely detection of emerging resistance in clinical *A. baumannii* isolates, ultimately informing treatment strategies and guiding future drug development.

## INTRODUCTION

*Acinetobacter baumannii* is a leading cause of hospital-acquired infections, with treatment options often limited due to multidrug resistance. In particular, the emergence and global spread of carbapenem-resistant *A. baumannii* (CRAb) represents a growing global threat, with reported 30-day mortality rates exceeding 20% across infection types (1). The World Health Organization (WHO) designated CRAb as a critical priority pathogen in its first global priority list of antibiotic-resistant bacteria (2) and reaffirmed this designation in the most recent update (3), underscoring the urgent need for new therapeutic strategies.

Cefiderocol is a novel chloro-catechol-conjugated siderophore cephalosporin developed by Shionogi & Co., Ltd. (Osaka, Japan) as a novel agent against multidrug-resistant gram-negative bacterial infections (4). Across studies of carbapenemase-producing strains, cefiderocol retained activity against more than 80% of isolates, including Enterobacterales (83%–90%) (5–8), *Pseudomonas aeruginosa* (96%–98.2%) (7–10), and CRAb (91.2%–97.9%) (6, 8, 11), according to the CLSI breakpoints (12). The R2 side chain of cefiderocol contains a catechol moiety that enables chelation of ferric ions, whereas the cephem core and the R1 side chain at the C7 position are structurally related to those of ceftazidime. This unique modification in the R2 side chain allows cefiderocol to exploit bacterial iron uptake systems for cell entry, thereby circumventing resistance mechanisms associated with outer membrane impermeability, such as porin loss and active efflux. Accordingly, amino acid substitutions leading to the loss of function of TonB-dependent receptors, which are involved in the uptake and transport of large substrates such as iron-siderophore complexes, including CirA in Enterobacterales, PiuA/PiuD in *P. aeruginosa*, and PiuA/PirA in *A. baumannii*, have been reported as major causes of cefiderocol resistance in clinical isolates (13–17). In addition to such permeability-related mechanisms, β-lactamase-mediated resistance to cefiderocol has been increasingly recognized. In particular, clinical isolates producing KPC variants demonstrating ceftazidime-avibactam resistance, the class A extended-spectrum β-lactamase (ESBL) PER-2, and cefepime-hydrolyzing class C β-lactamases (extended-spectrum AmpC, ESAC) have all been reported to confer resistance to cefiderocol (8, 18). Clinical isolates producing the class B metallo-β-lactamase NDM have also been reported to exhibit resistance (8); importantly, NDM efficiently hydrolyzes cefiderocol, whereas VIM and IMP mainly bind the drug without effective hydrolysis (19). Additionally, modifications in penicillin-binding protein (PBP) 3, particularly the four-amino acid insertions YRIN or YRIK, have been reported in some clinical isolates exhibiting reduced susceptibility to PBP3-targeting β-lactams, including cefiderocol (20, 21). The CREDIBLE-CR trial reported increased mortality among patients with CRAb infections treated with cefiderocol compared with those who received the best available therapy (22). Although baseline differences may partly explain this imbalance, heteroresistance among infecting CRAb isolates has also been proposed as a contributing factor (23–25). *A. baumannii* intrinsically harbors two chromosomal β-lactamases: the class C *Acinetobacter*-derived cephalosporinases (ADCs) and the class D OXA-51-group enzymes. Recent large-scale genomic surveys have revealed remarkable allelic diversity within these intrinsic β-lactamases, including more than 200 assigned and over 700 unassigned *bla*_ADC_ alleles, as well as a similar extent of diversity among intrinsic *bla*_OXA-51_ alleles (26). Although both intrinsic and acquired OXA-type enzymes appear to play only a limited role in cefiderocol resistance (27–29), ADCs have been implicated as potential contributors. In particular, ADC variants such as ADC-33, which carries an alanine duplication (ADUP) at position 218a in the Ω loop (residue numbering according to SANC [30]), exhibit enhanced hydrolytic activity against cefiderocol (31, 32). Moreover, the wide allelic diversity of ADCs, including rare variants, raises concern that some may further compromise cefiderocol activity. However, systematic investigation has not yet been undertaken to define and potentially predict which ADC variants contribute to cefiderocol resistance in *A. baumannii* clinical isolates.

To address this gap, we analyzed ADC variants from a large collection of CRAb clinical isolates and investigated the molecular mechanisms underlying cefiderocol resistance using susceptibility testing, mutagenesis, enzymatic kinetics, and crystallographic analysis.

## RESULTS AND DISCUSSION

### CRAb clinical strains harboring ADCs with both a Val292 substitution in the R2 loop and an ADUP in the Ω loop exhibit consistently high cefiderocol MICs

**Fig 1:**
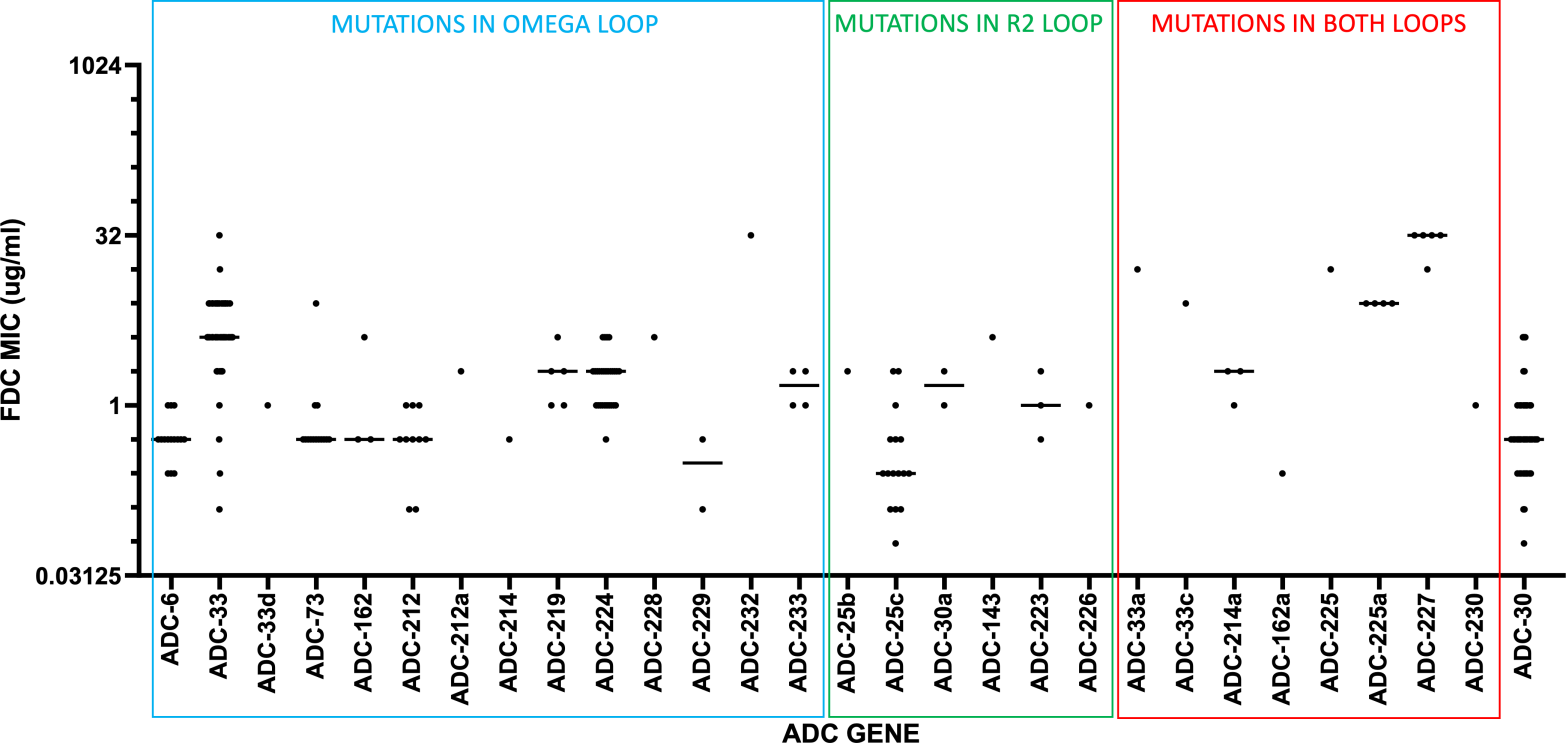
MIC profiles of cefiderocol among ADC variants identified in CRAb clinical isolates. Scatter plot showing the distribution of cefiderocol MICs for each ADC variant. The *x*-axis represents individual ADC variants, and the *y*-axis represents the corresponding MIC values (µg/mL) plotted on a log_2_ scale. Each dot corresponds to a single isolate. The horizontal line indicates the median MIC value.

A total of 428 CRAb isolates were obtained from the Study Network of *Acinetobacter* as a Carbapenem-Resistant Pathogen (SNAP). Isolates from this network were collected from hospitals across the United States between 2017 and 2019. These isolates harbored 52 distinct ADC variants, 28 of which carried variations in the R2 and/or Ω loop regions, including 6 variants in the R2 loop, 14 in the Ω loop, and 8 in both regions (Table S1). Susceptibility of these isolates to cefiderocol was tested; the results are summarized in Fig. 1. The results showed that while most variants exhibited MIC values of 0.5–2 µg/mL, the ADC-30 variants ADC-33, ADC-225, and ADC-227 exhibited elevated median MICs of 4, 8, and 32 µg/mL, respectively. ADC-33 and its related variants share an ADUP at position 218a and two amino acid substitutions (P213R and T320N) compared with ADC-30. It has been reported that ADC-33 confers reduced susceptibility to cefiderocol, primarily due to the ADUP (31, 32). ADC-225 is a variant of ADC-33, possessing two amino acid substitutions, D217N and V292G. ADC-227 possesses four amino acid substitutions (P213R, S232K, V292W, and K361E) in addition to an ADUP at position 218a, relative to the ADC-30 sequence.

In this collection, ADC-33 was the most common variant carrying substitutions in the Ω loop; however, isolates producing ADC-33 displayed a broad MIC distribution ranging from 0.13 to 32 µg/mL. In contrast, ADC-225 and ADC-227 carried valine substitutions at position 292 in the R2 loop (V292G in ADC-225, and V292W in ADC-227), in addition to the ADUP at position 218a observed in ADC-33. These isolates exhibited consistently high MICs, although the number of isolates was smaller than that of those harboring ADC-33, making it difficult to capture the full range of variation. Genomic analysis revealed no mutations in PBP3 or truncations in known proteins involved in cefiderocol resistance, such as PirA, PiuA, PiuC, or CarO, in the clinical isolates harboring ADC-227 (Table S2). These findings suggest that R2 loop substitutions, in addition to the ADUP at position 218a, may be associated with consistently high cefiderocol MICs. In particular, ADC-162a and ADC-214a, which possess the V292G substitution without the ADUP, were associated with MICs of 0.25 and 2 µg/mL, respectively, similar to those of most ADC variants. These results support the observation that variants such as ADC-225 or ADC-227, possessing both the Val292 substitution in the R2 loop and the ADUP in the Ω loop, are associated with consistently high cefiderocol MICs.

### ADC-227 confers high-level resistance to ceftazidime-avibactam and reduced susceptibility to cefiderocol

The Val292 residue is part of the substrate-binding pocket of the ADC enzyme. Considering the side chain volume of the substituted residues, the tryptophan substitution in ADC-227 is likely to have a greater impact on enzymatic activity due to steric hindrance within the pocket, whereas the glycine substitution in ADC-225 is expected to have a minimal effect. To examine the phenotype conferred by ADC-227, the *bla*_ADC_ gene was cloned into the cloning vector pBC SK(−), transformed into *E. coli* TOP10, and the susceptibility of the *E. coli* to cefiderocol, ceftazidime, and ceftazidime-avibactam was tested. These results demonstrated that ADC-33, ADC-225, and ADC-227 conferred reduced susceptibility to cefiderocol as expected. ADC-227 conferred greater resistance to ceftazidime-avibactam than that conferred by ADC-33 and ADC-225 (Table 1).

**TABLE 1:** MICs of *E. coli* TOP10 strains producing the ADC variant enzymes^*a*^

ADC variant	MIC (µg/mL)		
	CAZ	CZA	FDC
ADC-30	32	0.25	0.06
ADC-33	>64	4	1
ADC-225	64	4	1
ADC-227	>64	64	2
Control [pBC SK(−)]	0.25	0.25	0.06

^*a*^ CAZ, ceftazidime; CZA, ceftazidime/avibactam; FDC, cefiderocol; MIC, minimum inhibitory concentration.

Next, V292W substitutions were introduced into ADC-30 and ADC-33, respectively, and susceptibility to ceftazidime-avibactam was tested. In this experiment, the strains harboring ADC-30_V292W and ADC-33_V292W exhibited 32-fold and 128-fold higher MICs of 16 and 64 µg/mL, respectively, relative to the control strain, whose MIC was 0.5 µg/mL. In addition, we tested susceptibility to cefiderocol using the Shionogi MIC Dry Plate Cefiderocol (Kyokuto Pharmaceutical Industrial Co., Ltd., Tokyo, Japan). The MICs of strains harboring the control pBC SK(−), ADC-33, and ADC-227 were ≤0.03, 0.12, and 0.5 µg/mL, respectively. The strains harboring ADC-30_V292W and ADC-33_V292W exhibited more than 2-fold and 16-fold higher MICs of 0.06 and 0.5 µg/mL, respectively, relative to the control strain. These results indicate that the V292W substitution in ADC-227 is critical for conferring resistance to ceftazidime-avibactam and reduced susceptibility to cefiderocol. We previously reported a similar alteration of the susceptibility profile conferred by a plasmid-mediated AmpC, CMY-185, which is a CMY-2 variant possessing four amino acid substitutions (A114E, Q120K, V211S, and N346Y) (33, 34).

In CMY-185, the substituted Tyr346 residue functions as a substrate sensor that distinguishes cephalosporins from avibactam based on their interaction patterns; it accommodates cephalosporins through a drastic structural change of the R2 loop, while strongly rejecting avibactam binding (34). The substituted Ser211 residue contributes to the rapid release of the product by enhancing the turnover rate of ceftazidime and cefiderocol. The characteristics of these substitutions in ADC-227 and CMY-185 are similar: both enzymes harbor substitutions to bulkier residues in the R2 loop, while substitution or insertion in the Ω loop enhances the enzymatic turnover rate. These findings suggest a potent molecular mechanism conferred by the V292W substitution in ADC-227.

### ADC-227 exhibits increased catalytic efficiencies for cephalosporins and a moderate reduction in avibactam inhibition

To elucidate the molecular mechanism conferred by the V292W substitution, the steady-state kinetic parameters *k*_cat_ and *K*_*m*_ for nitrocefin, cephalothin, ceftazidime, and cefiderocol, as well as the inhibition parameters *K*_*i* app_, *k*_2_/*K*, *k*_−2_, and *k*_off_ for avibactam, were determined (Table 2). The results showed that ADC-33 and ADC-227 possess broad substrate specificities, including activity against cefiderocol, whereas ADC-30 displays superior activity against more classical cephalosporin substrates such as cephalothin and nitrocefin. Compared with ADC-30 and ADC-33, ADC-227 exhibited lower *K*_*m*_ values for all tested cephalosporins. Although the turnover rates (*k*_cat_) of ADC-227 were also lower for all tested cephalosporins than those of ADC-33, the overall catalytic efficiencies (*k*_cat_/*K*_*m*_) for ceftazidime and cefiderocol were elevated due to reduced *K*_*m*_ values, while those for cephalothin and nitrocefin were comparable. For AmpC β-lactamase, deacylation is considered to be a rate-limiting step for the third-generation cephalosporins (35, 36). Therefore, these results are consistent with reduced turnover and stabilization of the acyl-enzyme complex state resulting from a slower deacylation step in ADC-227. In addition, for ceftazidime and cefiderocol, elevated *k*_cat_/*K*_*m*_ values indicate greater catalytic efficiency at low substrate concentrations, highlighting that the balance between catalytic efficiency and turnover is a critical determinant of the resistance phenotype. The V292W substitution appears to modulate the catalytic behavior of ADCs by reducing turnover while increasing overall catalytic efficiency for cephalosporins, including cefiderocol. Although the MIC shift observed in *E. coli* TOP10 recombinants was subtle when comparing the strain harboring ADC-227 with that carrying ADC-33 (Table 1), the ADC-227 variant in CRAb clinical isolates exhibited a consistently high median cefiderocol MIC of 32 µg/mL (Fig. 1). This finding suggests that the impact of this substitution becomes more pronounced in the native host background, where permeability and efflux also influence drug exposure.

**TABLE 2:** Kinetic and inhibition parameters of ADC-30, ADC-33, and ADC-227^*a*^

Substrate and kinetic parameter	ADC-30	ADC-33	ADC-227
Nitrocefin			
*k*_cat_ (×10^3^ s^−1^)	5.5 ± 0.7	2.4 ± 0.2	0.80 ± 0.04
*K*_*m*_ (µM)	194.2 ± 31.3	145.1 ± 13.7	47.3 ± 3.6
*k*_cat_/*K*_*m*_ (µM^−1^ s^−1^)	28.2 ± 5.8	16.9 ± 2.1	17.0 ± 1.5
Cephalothin			
*k*_cat_ (×10^3^ s^−1^)	22.6 ± 0.3	0.31 ± 0.01	0.22 ± 0.00
*K*_*m*_ (µM)	691.1 ± 110.0	40.9 ± 1.9	30.5 ± 0.5
*k*_cat_/*K*_*m*_ (µM^−1^ s^−1^)	32.7 ± 7.2	7.6 ± 0.4	7.4 ± 0.1
Ceftazidime			
*k*_cat_ (s^−1^)	0.28 ± 0.03^*b*^	2.2 ± 0.1	0.47 ± 0.01
*K*_*m*_ (µM)	31.8 ± 5.0^*b*^	23.9 ± 1.5	1.7 ± 0.2
*K*_i_ (µM)	8.0 ± 1.3^*b*^	N.C.	N.C.
*k*_cat_/*K*_*m*_ (µM^−1^ s^−1^)	0.01 ± 0.00^*b*^	0.09 ± 0.01	0.29 ± 0.03
Cefiderocol			
*k*_cat_ (s^−1^)	0.003 ± 0.000	0.35 ± 0.02	0.17 ± 0.02
*K*_*m*_ (µM)	13.6 ± 4.3	123.6 ± 12.7	7.8 ± 1.6
*k*_cat_/*K*_*m*_ (×10^−2^ µM^−1^ s^−1^)	0.02 ± 0.01	0.28 ± 0.03	2.2 ± 0.5
Avibactam			
*K*_*i* app_ (µM)	N.D.	25.2 ± 1.4	89.6 ± 9.9
*k*_2_/*K* (M^−1^ s^−1^)	N.D.	349.0 ± 8.0	186.1 ± 17.4
*k*_−2_ (×10^−4^ s^−1^)	N.D.	44.5 ± 1.4	0.0 ± 4.0^*c*^
*k*_off_ (×10^−6^ s^−1^)	N.D.	10.8 ± 0.0	5.2 ± 0.0

^*a*^ The values are the mean ± standard error of three independent measurements. N.D. = not determined; N.C. = not calculated.

^*b*^ Due to substrate inhibition, the kinetic parameters for ceftazidime with ADC-30 were determined using the Haldane equation (37).

^*c*^ *k*_−2_ for ADC-227 was below the detection limit and is reported as ~0.

To measure the inhibition parameters of avibactam, nitrocefin and cephalothin were deemed unsuitable as reporter substrates for ADC-30 due to their high *K*_*m*_ values. Accordingly, measurements were limited to ADC-33 and ADC-227. The apparent inhibitory constants (*K*_*i* app_) of avibactam for ADC-33 and ADC-227 were 25.2 ± 1.4 and 89.6 ± 9.9 µM, respectively, indicating reduced inhibition of ADC-227 by avibactam. Avibactam is a covalent, reversible inhibitor that binds to the enzyme, carbamoylates the active-site serine residue, and is subsequently released through decarbamoylation via hydrolysis or intramolecular ring closure, thereby regenerating free avibactam (38). Detailed kinetic analysis of binding and dissociation revealed that the *k*_₋2_ value, representing the rate of avibactam regeneration, is more than 10-fold higher for ADC-33 than for ADC-227, while both the carbamoylation (*k*_2_/*K*) and the decarbamoylation (*k*_off_) rates are approximately 2-fold higher in ADC-33. These results indicate that the V292W substitution confers a moderate reduction in avibactam inhibition. The impact of the Val292 substitution on the reduction of avibactam inhibition was less pronounced than anticipated. In comparison, the *K*_*i* app_ of avibactam for CMY-2_N346Y is 1,000-fold higher than that of CMY-2 (34). However, the absolute *K*_*i* app_ values of ADC-33, ADC-227, and CMY-2 variants carrying the N346Y substitution are at or above the avibactam concentration of 14 µM (≈4 µg/mL) used in susceptibility testing, suggesting that the amount of avibactam in standard assays may be insufficient to fully inhibit these enzymes. Consistently, the strain producing ADC-33 also exhibited reduced susceptibility to ceftazidime-avibactam relative to the control strain (Table 1). Taken together, these findings indicate that ADC-227 acquires high-level resistance to ceftazidime-avibactam primarily through enhanced ceftazidime hydrolysis, in combination with moderately reduced avibactam inhibition. They also suggest that functional consequences of bulky substitutions within the R2 loop may vary depending on their exact position.

### Crystal structures of ADC-227 and overall structure comparisons

The kinetic analysis of ADC-227 exhibited its increased catalytic efficiencies for ceftazidime and cefiderocol and reduced inhibition by avibactam, resulting in resistance to ceftazidime-avibactam and reduced susceptibility to cefiderocol. Previously, we reported the first cases of cefiderocol resistance mediated by an AmpC enzyme (39, 40). In particular, AmpC^Ent385^ exhibits higher catalytic efficiency for cefiderocol and confers reduced susceptibility due to the deletion of alanine-proline residues at positions 294–295 in the R2 loop, which expands the substrate-binding site (39). Compared with typical AmpC enzymes such as P99, almost all ADCs inherently possess a three-amino acid deletion at positions 304–306 in the R2 loop (41), suggesting that ADCs have a general tendency to exhibit higher efficiency toward cefiderocol. However, the V292W substitution in ADC-227 may further enhance this efficiency, prompting further structural analysis of ADC-227.

Crystals of ADC-227 in the free form were prepared, and the structure was determined at 1.95 Å resolution. Attempts to obtain crystals of the ADC-227-ceftazidime complex, whose acyl-enzyme intermediate is essentially identical to that of the ADC-227-cefiderocol complex, using a soaking procedure, were unsuccessful. Given that our kinetic studies indicated moderate affinity of avibactam for ADC-227, and considering that avibactam is smaller in molecular size than ceftazidime, attempts were made to obtain crystals of the ADC-227-avibactam complex. Crystals of the ADC-227-avibactam complex were successfully obtained by soaking free-form crystals in mother liquor containing either 50 or 100 mM avibactam for either 4 or 24 h, yielding four distinct conditions for crystallographic analysis. In this study, three crystal structures of the ADC-227-avibactam complex were determined under the following conditions: 50 mM for 4 h, 50 mM for 24 h, and 100 mM for 4 h, at resolutions of 1.90, 2.10, and 2.11 Å, respectively. Data collection and refinement statistics are summarized in Table S3. Crystallographic analysis revealed that ADC-227 crystallized in two distinct space groups depending on the avibactam incubation conditions. The free enzyme and one avibactam complex, which was obtained under 50 mM for 4 h, belonged to space group *P*3_1_21 and contained one monomer in the asymmetric unit, whereas the remaining avibactam complexes crystallized in space group *P*3_2_21 with two monomers in the asymmetric unit (Table S3). In total, six structures of ADC-227 were determined, including one free form and five avibactam-bound complexes.

**Fig 2:**
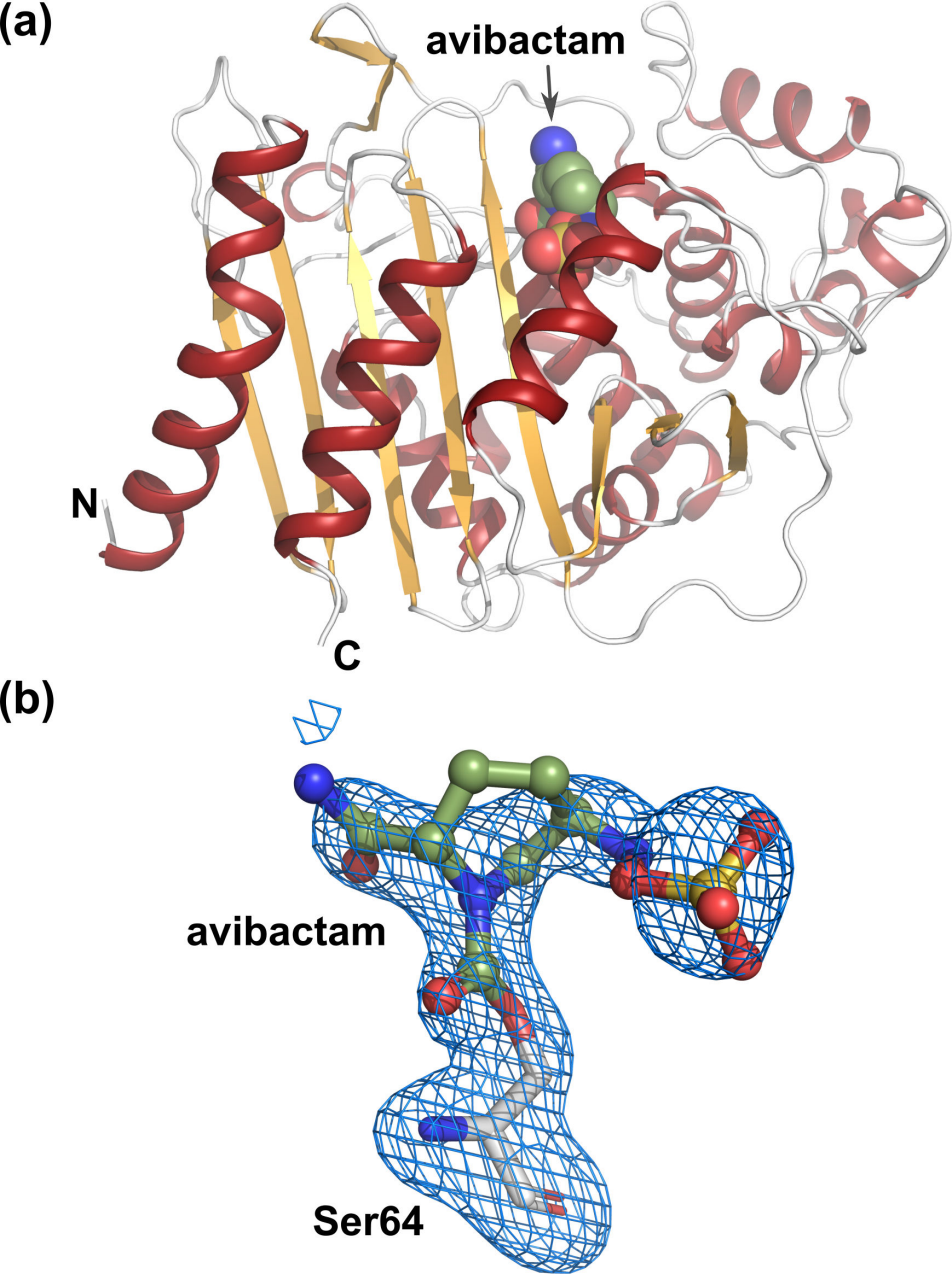
Overall structure of ADC-227. (**a**) The ADC-227-avibactam complex was obtained under the condition of 50 mM for 4 h. (**b**) Close-up view of the avibactam molecule. The avibactam molecule is shown as a ball-and-stick representation colored green. The m*F*_o_-D*F*_c_ polder omit map is shown as a blue mesh contoured 4σ.

**Fig 3:**
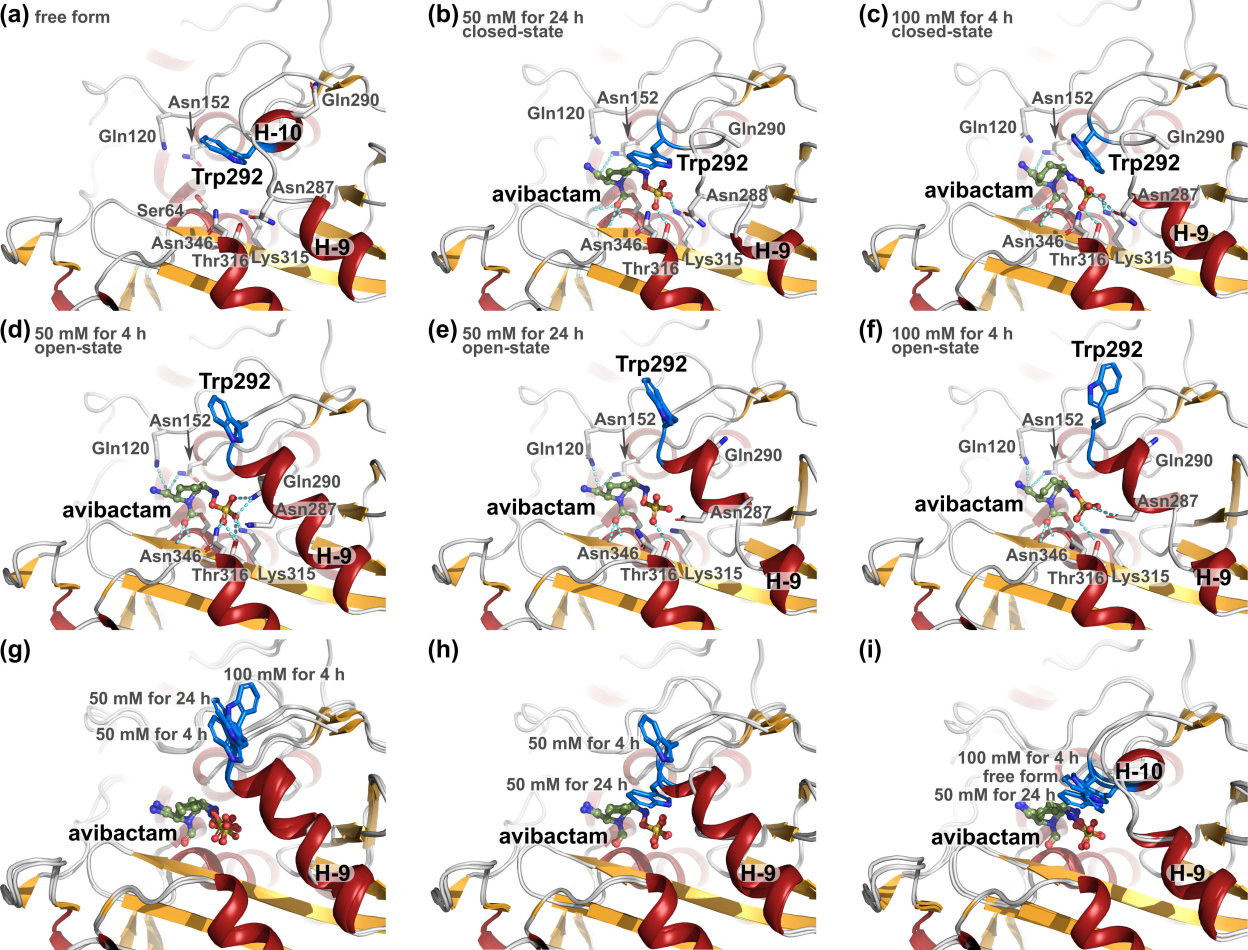
Structure comparisons of ADC-227. The avibactam molecule is shown as a ball-and-stick representation in green. Trp292 is shown as a blue stick. Hydrogen bonds are indicated by cyan dashed lines. (**a**) Free form. (**b, e**) The avibactam complex was obtained under the condition of 50 mM for 24 h. (**c, f**) The avibactam complex was obtained under the condition of 100 mM for 4 h. (**d**) The avibactam complex was obtained under the condition of 50 mM for 4 h. (**g**) Structural comparison of the open state. (**h**) Structural comparison of the open and closed states. (**i**) Structural comparison of the closed state.

The overall structures of ADC-227 were similar to one another, with rmsd values of 0.26–0.32 Å for the Cα positions of residues 287–311 (Fig. 2a). They were also comparable to the structure of ADC-33 in the free form (PDB ID: 8FQN (31)), with rmsd values of 0.27–0.46 Å for the Cα positions of residues 284–307. The binding position of avibactam was identical among the ADC-227-avibactam complex structures. The electron density maps were continuously extended to the catalytic Ser64 residue of ADC-227, indicating that these structures represent covalent carbamoyl-enzyme complexes (Fig. 2b). However, the conformation of the R2 loop and the hydrogen-bonding network involved in avibactam recognition differed among the ADC-227 complex structures (Fig. 3 and Fig. S1).

### Crystallographic analysis of ADC-227 captured a transient structure during substrate recognition

In ADC-227, compared with the free form, the avibactam complex obtained under the condition of 50 mM for 4 h revealed a drastic structural rearrangement of the R2 loop, in which the H-10 helix fused with the H-9 helix to form a single extended α-helix, and the substituted Trp292 residue was flipped outward, positioned outside the substrate-binding pocket (Fig. 3a and d). Typically, AmpC enzymes recognize avibactam through a tight hydrogen-bonding network: Gln120 and Asn152 form hydrogen bonds with the amide group on the piperidine ring of avibactam, while Asn289, Lys315, Thr316, and Asn346 interact with the sulfate group of avibactam (39, 42–44). The hydrogen-bonding network involved in avibactam recognition in this structure of the ADC-227-avibactam complex was conserved and resembled that of typical AmpC enzymes (Fig. 3d).

The crystal structure of the ADC-227-avibactam complex obtained under the condition of 50 mM for 24 h contained two monomers of ADC-227. These monomers adopted two distinct conformations: one represented the open-state structure, similar to that observed under the condition of 50 mM for 4 h (Fig. 3e), while the other represented a closed-state structure. In the closed state, the R2 loop underwent a structural rearrangement in which the H-9 and H-10 helices were separated, resembling the organization seen in the free-form structure (Fig. 3h and i). In this closed conformation, the substituted Trp292 residue was reoriented to lie over the substrate-binding pocket, covering it from above (Fig. 3b). The hydrogen-bonding network involved in avibactam recognition also underwent rearrangement (Fig. 3b). In the open-state structure, hydrogen bonds between the sulfate group of avibactam and Gln290, Lys315, and Asn346 were disrupted, whereas the amide group of avibactam maintained hydrogen bonds with Gln120 and Asn152 (Fig. 3e). Compared with the open-state structure, Lys315 re-formed a hydrogen bond with the sulfate group, while the hydrogen bond between Gln120 and the amide group was disrupted in the closed-state structure (Fig. 3b). A similar structural rearrangement was observed in the ADC-227-avibactam complex obtained under the 100 mM for 4 h condition, except that Lys315 retained and Asn287 newly formed hydrogen bonds with the sulfate group of avibactam in both the open and closed states (Fig. 3c and f). These observations indicate conformational flexibility of the R2 loop and plasticity at the ADC-227-avibactam interface. In particular, this interfacial flexibility may account for the lower apparent affinity of avibactam for ADC-227 relative to other AmpC enzymes (34, 45–47) and the correspondingly reduced extent of inhibition.

Other crystal structures of the ADC-227-avibactam complex, for which data sets were collected at the same time as those described above, were not refined to completion. These structures showed that the main chain of the R2 loop regions was largely disordered, likely reflecting structural heterogeneity within the crystals, including possible resolution limitations, making them unsuitable for detailed discussion. Nevertheless, based on their space groups, the crystal structures could be categorized as representing either the open state or a mixture of the open and closed state, as follows: conditions (mixed-state crystals/total number of crystals collected data set), 50 mM for 4 h (0/7), 50 mM for 24 h (3/6), 100 mM for 4 h (3/4), and 100 mM for 24 h (3/3). These results indicate that the proportion of the mixed state increases with higher avibactam concentrations or longer soaking times and that the R2 loop of ADC-227 transiently adopts an open state to accommodate avibactam before shifting to a closed state. The driving force for the adaptation of the open-state structure could be explained by steric hindrance between Trp292 and the binding substrate. The driving force for shifting to the closed-state structure after substrate binding is considered to involve two possibilities: one is the rearrangement of the hydrogen-bond network involved in avibactam recognition, and the other is stabilization by crystal packing, which favors the closed-state conformation. In fact, we were unable to obtain the ADC-227-ceftazidime complex and believe that this failure was likely due to crystal packing, which may not allow the structural rearrangements of the R2 loop required for ceftazidime binding. This observation is consistent with the previous discussion on CMY-185, in which it was proposed that the order of recognition differs between cephalosporins and avibactam (34). Specifically, cephalosporins are recognized in a more global manner, with their R1 side chain and cephem core structure first accommodated, followed by recognition of the R2 side chain by the R2 loop, whereas avibactam enters the binding pocket by first inserting its sulfate group, which is recognized by the R2 loop.

**Fig 4:**
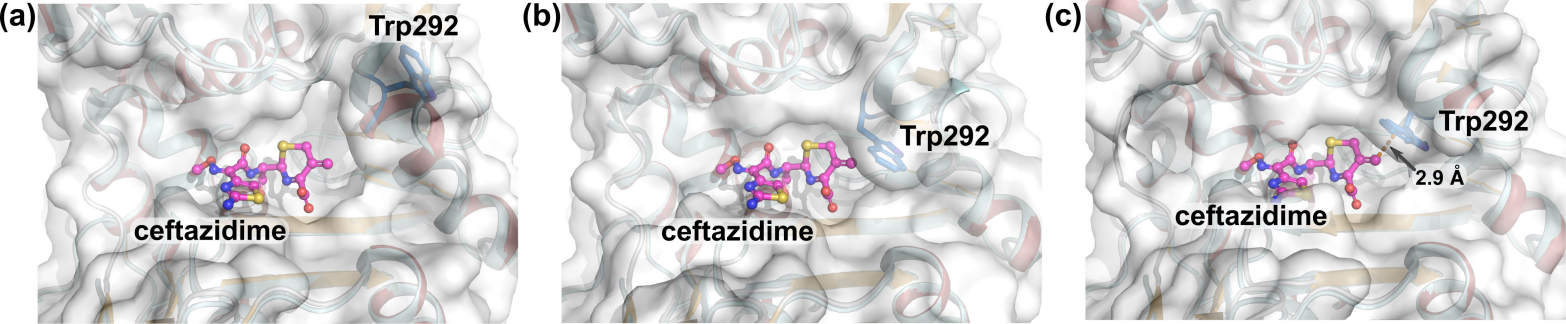
Model structures of the ADC-227–ceftazidime complex. The ceftazidime molecule is shown in magenta using a ball-and-stick representation. Trp292 is depicted as blue sticks. The crystal structure of the ADC-33-ceftazidime complex is shown as a cyan cartoon representation. Transparent molecular surfaces of each state of ADC-227 are colored in white. (**a**) Open state. (**b**) Closed state derived from the ADC-227-avibactam complex. (**c**) Closed state derived from the free-form structure of ADC-227. Orange-dashed lines indicate the distance between the centroid of the six-membered ring of the indole moiety of Trp292 and the methylene group at the C3 position of ceftazidime.

Furthermore, model structures of the ADC-227-ceftazidime complex were generated by superposition of the ADC-227-avibactam structures onto the crystal structure of the ADC-33-ceftazidime complex (PDB ID: 9EHY [32]) (Fig. 4). The open-state conformation could accommodate ceftazidime without steric clashes (Fig. 4a). In contrast, the closed-state models derived from the avibactam complex appeared unstable due to steric interactions between the Cβ atom of Trp292 and either the C2 atom or the methylene group at the C3 position of ceftazidime (Fig. 4b). However, it is conceivable that ceftazidime may adopt a slightly shifted orientation in the actual complex to mitigate these steric constraints. Consistent with this notion, the model derived from the free-form structure of ADC-227 allowed ceftazidime accommodation, stabilized by a CH–π interaction between the indole ring of Trp292 and the methylene group of ceftazidime (Fig. 4c). Taken together, these observations suggest that even in the ceftazidime or cefiderocol complexes, ADC-227 may initially adopt an open-state conformation to accommodate the cephalosporins, followed by a conformational change in which Trp292 acts as a lid to stabilize the binding.

### ADC-227 does not appear to confer resistance to carbapenems or sulbactam-durlobactam

Resistance to carbapenems in *A. baumannii* has generally been attributed to OXA-type class D β-lactamase, including acquired enzymes such as OXA-23 and OXA-58, as well as IS*AbaI*-driven overexpression of the intrinsic OXA-51-group enzymes (48). Recently, Stewart et al. reported that ADC-1_TM_, an ADC-1 variant possessing three amino acid substitutions (V292F, S318T, and F322S), conferred carbapenem resistance to its *A. baumannii* transformant (49). This finding suggested that ADC-227 might also confer carbapenem resistance, since both ADC-227 and ADC-1_TM_ share a common substitution of valine to an aromatic amino acid at position 292, and even the ADC-1 variant possessing only the V292F substitution was shown to confer reduced susceptibility to carbapenems (48). In particular, crystallographic analysis of ADC-227 revealed that the Trp292 residue acts as a lid covering the substrate-binding pocket from above; a similar molecular mechanism is observed in the KPC-2 carbapenemase (50, 51). In KPC-2, Trp105 is crucial for enzymatic activity against both carbapenems and cephalosporins, and substitutions at this position with non-aromatic residues markedly reduce their activity (50, 51). Thus, the susceptibility of *E. coli* TOP10 transformants harboring ADC-30, ADC-33, ADC-227, ADC-30_V292W, and ADC-33_V292W to ertapenem and meropenem was tested. All transformants exhibited comparable MIC values to those of the control strain harboring pBC SK(−), with the values of 0.016 µg/mL for ertapenem and 0.06 µg/mL for meropenem. Previous results suggesting carbapenem resistance were obtained using an *A. baumannii* transformant (49). Therefore, we next generated *Acinetobacter baylyi* recombinant strains ADP1_adc-30, ADP1_adc-33, and ADP1_adc-227, in which the intrinsic *bla*_ADC_ gene was replaced by *bla*_ADC-30_, *bla*_ADC-33_, and *bla*_ADC-227_, respectively, via recombination. ADP1_adc-33 and ADP1_adc-227 exhibited 4-fold and 16-fold increased MICs of cefiderocol relative to ADP1_parent and ADP1_adc-30 (Table 3). However, ADP1_adc-227 exhibited only a 2-fold increase in MICs, with values of 2 µg/mL for ertapenem and 0.13 µg/mL for meropenem, compared with 1 µg/mL and 0.06 µg/mL, respectively, for the ADP1_parent strain (Table 3). These results indicate that ADC-227 is unlikely to confer significant carbapenem resistance. Several explanations are possible: ADC-1 possesses two substitutions, T316N and G317R, relative to ADC-30, and these residues, located near the substrate-binding pocket, may contribute to carbapenem resistance. In contrast, the ADUP at position 218a in ADC-227 might influence Ω-loop dynamics in a manner that is advantageous for cephalosporin release but unfavorable for carbapenem hydrolysis, thereby interfering with resistance. Alternatively, the phenotype may be specific to *A. baumannii* and absent in *A. baylyi*. At present, the precise reason remains unclear, and further studies will be required to resolve this discrepancy.

**TABLE 3:** MICs of *A. baylyi* ADP1 recombinant strains producing ADC variant enzymes^*a*^

ADC variant	MIC (µg/mL)					
	FDC	ETP	MEM	SUL	DUR	SUL–DUR
ADC-30	0.12	1	0.06	1	8	0.12
ADC-33	0.5	1	0.06	1	4	0.12
ADC-227	2	2	0.12	2	16	0.25
ADP1_Parent	0.12	1	0.06	2	16	0.25
*E. coli* ATCC25922	0.5	<0.12	<0.03	32	0.25	N.G.

^*a*^ DUR, durlobactam; ETP, ertapenem; FDC, cefiderocol; MEM, meropenem; SUL, sulbactam; SUL–DUR, sulbactam–dulrobactam; MIC, minimum inhibitory concentration; N.G., No growth.

Sulbactam-durlobactam is a novel β-lactamase inhibitor combination approved by the U.S. Food and Drug Administration (FDA) in 2023 for the treatment of hospital-acquired and ventilator-associated bacterial pneumonia caused by *A. baumannii* in adults. We previously evaluated the susceptibility of CRAb isolates from the SNAP study to sulbactam-durlobactam and reported that A515V or T526S substitutions in PBP3 are associated with reduced susceptibility (52). In our earlier analysis, the clinical isolates harboring ADC-227, which were the focus of the present study, were also included. These five isolates were collected at a single hospital in the United States and showed MIC values of 1 µg/mL (*n* = 1), 4 µg/mL (*n* = 3), and 8 µg/mL (*n* = 1) without PBP3 substitutions, suggesting that ADC-227 could be associated with a modest trend toward reduced susceptibility to sulbactam-durlobactam. To further examine, we tested sulbactam-durlobactam susceptibility in ADP1_adc-30, ADP1_adc-33, and ADP1_adc-227. The MIC values for these strains were comparable to those of the ADP1_parent strain (Table 3), indicating that ADC enzymes do not confer reduced susceptibility to sulbactam-durlobactam.

### Conclusion

Here, we describe significant variations in cefiderocol susceptibility among CRAb clinical isolates. A comprehensive analysis of clinical isolates, mutagenesis, enzymatic kinetics, and crystallography demonstrates that stable cefiderocol resistance requires the combination of a Val292 substitution in the R2 loop and an ADUP at position 218a in the Ω loop. In particular, the ADC-227 variant harboring a V292W substitution confers not only reduced susceptibility to cefiderocol but also high-level resistance to ceftazidime-avibactam, primarily due to enhanced hydrolysis of ceftazidime. This phenotype arises from a trade-off between increased catalytic efficiency for ceftazidime and cefiderocol and moderately reduced avibactam inhibition, together with conformational flexibility of the R2 loop. Taken together with the sulbactam-durlobactam susceptibility data, the present findings do not support a generalized resistance mechanism against diazabicyclooctane-based β-lactamase inhibitors. Collectively, the findings identify Val292, in combination with an ADUP in the Ω loop, as a mutational “hot spot” for resistance evolution in ADCs: substitutions at this position, whether to small or bulky residues, consistently enhance cefiderocol resistance and, in the case of substitution with tryptophan, also drive resistance to ceftazidime-avibactam. Although ADC-227 was not associated with carbapenem resistance in the described assays, related variants carrying an aromatic substitution at this position have been implicated in carbapenem resistance. The future emergence of Val292 substitutions may therefore compromise the efficacy of multiple β-lactam agents. Together, these findings highlight the potential for ADC evolution to undermine newer β-lactams, underscoring the importance of routine surveillance and molecular characterization of *A. baumannii* isolates in clinical settings.

## MATERIALS AND METHODS

### Cloning, expression, and purification of the ADC-30 variants

For ADCs cloning, genomic DNA was extracted by heating at 95°C for 10 min, followed by centrifugation at 18,000 × *g* for 5 min. Each *bla*_ADC_ gene was amplified by PCR using the extracted genomic DNA as a template and cloned into the BamHI-HindIII-double-digested pET-24a(+) plasmid vector. For susceptibility testing, the *bla*_ADC_ _pET-24a(+) plasmid vector was then digested with restriction enzymes XbaI and HindIII, and the DNA fragment harboring *bla*_ADC_ was ligated into the XbaI-HindIII-double-digested pBC SK(–) plasmid vector. *E. coli* TOP10 cells were transformed with the ligation product, and transformants were selected by growth on lysogeny broth agar containing 100 µg/mL ampicillin and 30 µg/mL chloramphenicol. V292W substitutions in ADC-30 and ADC-33 were introduced by site-directed mutagenesis using corresponding *bla*_ADC__pBC SK plasmid vectors as the templates. DNA fragments containing the mutations and their corresponding reverse fragments were amplified by PCR, and the resulting overlapping fragments were assembled using the In-Fusion Snap Assembly Kit (Takara Bio, Japan). For kinetic analysis and crystallization, each *bla*_ADC_ gene lacking the signal peptide region was amplified by PCR using the corresponding *bla*_ADC__pET-24a(+) plasmid vector as a template and assembled with NdeI–EcoRI–double-digested pET-30b(+) plasmid vector using the In-Fusion Snap Assembly Kit (Takara, Shiga, Japan). All recombinant plasmids were sequenced on both strands by Sanger sequencing.

*E. coli* BL21(DE3) cells were transformed with the *bla*_ADC__pET-30b(+) plasmid vector, and expression and purification of all ADC-30 variants were performed using the previously reported procedures described for the CMY-2 variants (34).

### Chromosomal recombineering

We utilized a previously reported method of overlapping PCR and *A. baylyi*’s natural transformation ability to generate targeted chromosomal substitutions of the *bla*_ADC_ gene in *A. baylyi* with a few changes (53). First, four individual PCR reactions were run using Phusion Taq polymerase (Thermo Fisher Scientific, Waltham, MA, USA) to obtain fragments of DNA that contained ~500 bp upstream and downstream of the *bla*_ADC_ gene in *A. baylyi,* the targeted *bla*_ADC_ gene from clinical strains, and a selection cassette that contained a gentamycin marker. Each section contained approximately 20–30 bp of overhang with homology to the adjacent section. Primer sequences used to amplify each of these four sections are listed in Table S4. These four products were purified, and 30 ng of each was mixed in a 45 µL reaction containing polymerase and buffer. This reaction was run for 15 cycles in order to generate a full-length product. Afterward, the outer primers (All_ADCs_upstream_FOR and All_ADCs_downstream_REV) were added to amplify the full-length cassette for an additional 15 cycles. A full-length product was confirmed by agarose gel electrophoresis.

The remaining PCR reaction was added to 70 µL of an overnight *A. baylyi* culture grown in lysogeny broth and 1 mL of fresh media. This mixture was incubated overnight. The following day, recombinants were selected by plating 100 µL of culture onto lysogeny agar plates containing 10 µg/mL of gentamycin. Positive clones containing the *bla*_ADC_ gene of interest were confirmed by sequencing.

### Susceptibility testing

CRAb clinical isolates and *E. coli* TOP10 harboring the recombinant plasmids *bla*_ADC__pBCSK plasmid vector were grown on lysogenic broth agar plates at 37°C overnight, except that TOP10 cells were grown on the agar plates supplemented with 30 µg/mL chloramphenicol. MICs were determined in triplicate by the broth microdilution method using cation-adjusted Mueller-Hinton broth. Iron-depleted Mueller-Hinton broth was used for cefiderocol testing, following the guidelines established by the Clinical and Laboratory Standards Institute (CLSI) (54). To examine the effects of the V292W substitution in ADC-30 or ADC-33 on cefiderocol resistance, tests were also performed using the Shionogi MIC Dry Plate Cefiderocol (Kyokuto Pharmaceutical Industrial Co., Ltd., Tokyo, Japan), which is commercially available in Japan and was used due to the limited availability of iron-depleted Mueller-Hinton broth.

### Kinetic measurements

Steady-state kinetic and inhibition assays were conducted as described previously (34, 55). In brief, reactions were carried out in PBS (pH 7.2) at 25°C with a fixed enzyme concentration and varying substrate concentrations. Changes in absorbance associated with substrate hydrolysis were monitored using a UV-visible spectrophotometer (UV-1280, Shimadzu, Japan). Nitrocefin, cephalothin, ceftazidime, and cefiderocol were used as substrates, whereas avibactam was evaluated as an inhibitor. *K*_*m*_ and *k*_cat_ values were obtained by fitting the initial velocity data to the Michaelis-Menten equation. For competitive inhibition analyses, cephalothin (224 µM) was used as the reporter substrate, with enzyme concentrations ranging from 0.5 to 1 nM. Apparent *K*_*i* app_ values were calculated from Dixon plots by linear regression analysis. The observed *K*_*i* app_ values were corrected for substrate affinity according to equation 1 :


 (1) $$K_{i\text{ app}}=\frac{K_{i\text{ app (observed)}}}{1+\frac{\left[S\right]}{K_{m\text{(cephalothin)}}}}$$

where [*S*] represents the concentration of cephalothin and *K*_*m*_(cephalothin) denotes its Michaelis constant for each enzyme.

The dissociation rate constant (*k*_off_) was determined using a jump dilution approach. Enzyme-avibactam mixtures were preincubated at 25°C for 5 min with inhibitor concentrations approximately 10-fold higher than the corresponding *K*_i app_ (observed) values. The mixtures were subsequently diluted 2,000-fold into buffer containing 240 µM cephalothin, and recovery of enzymatic activity was monitored. All experiments were performed in triplicate. Curve fitting and regression analyses were conducted using R version 4.1.2 (R Core Team, 2021).

### Crystallization of ADC-227

Purified recombinant ADC-227 was prepared at 15 mg/mL in 20 mM Tris-HCl, pH 7.0, for crystallization. Crystals were grown at 20°C using the hanging-drop vapor diffusion method by mixing equal volumes of protein solution and reservoir solution containing 25% (wt/vol) PEG3350 and 0.1 M HEPES-NaOH, pH 7.0. To obtain the ADC-227-avibactam complex, ADC-227 crystals were soaked at 4°C for 4 or 24 h in reservoir solution supplemented with 50 or 100 mM avibactam.

### Data collection, structure determination, and refinement

Prior to data collection, ADC-227 crystals were cryoprotected in reservoir solution supplemented with 30% (vol/vol) glycerol and rapidly cooled in liquid nitrogen. Diffraction data were obtained at beamline BL-17A of the Photon Factory (High Energy Accelerator Research Organization, Tsukuba, Japan). Data were recorded at −173°C using an EIGER X16M detector and processed with XDS (56). The structure was solved by molecular replacement with Molrep (57) within the CCP4 suite (58), using the ADC-68 structure (PDB ID: 4QD4 [59]) as the search model. Model building and refinement were conducted with COOT (60) and phenix.refine from the PHENIX package (61), respectively. Refinement included translation/libration/screw (TLS) parameters, and TLS groups were identified using phenix.find_tls_groups. The stereochemical quality of the final model was assessed with MolProbity (62). Molecular graphics were generated using PyMOL (63).

## Data Availability

The atomic coordinates of the ADC-227 in the free form and in the avibactam complex have been submitted into the Protein Data Bank under PDB accession numbers 9WIP, 9WIQ, 9WIR, and 9WIS. Whole-genome sequencing data of the CRAb isolates analyzed in this study have been deposited in NCBI under BioProject accession number PRJNA906166.

## SUPPLEMENTAL MATERIAL

Supplemental material mbio.03518-25-s0001.pdf Tables S1, S3, and S4; Fig. S1.

Table S2 mbio.03518-25-s0002.xlsx Key genetic features of *A. baumannii* clinical isolates.

ASM does not own the copyrights to Supplemental Material that may be linked to, or accessed through, an article. The authors have granted ASM a non-exclusive, world-wide license to publish the Supplemental Material files. Please contact the corresponding author directly for reuse.
