# Supplementary material for: A Val292 substitution combined with an alanine duplication (ADUP) in the Ω loop of ADC β-lactamase confers reduced susceptibility to advanced β-lactam agents, including cefiderocol
Source: mBio. 2026 Apr 8;17(5):e03518-25. doi: 10.1128/mbio.03518-25 (PMC13170308; doi:10.1128/mbio.03518-25)
Supplement: Supplemental material — Tables S1, S3, and S4; Fig. S1. [file mbio.03518-25-s0001.pdf]

Table S1. Amino acid substitution profiles in the Ω and the R2 loops of ADC variants.

ADC variants	Ω loop	R2 loop
**Ω loop**		
ADC-6	Gly222Ser	
ADC-33	Pro213Arg; Ala218 dup (SANC 218a)	
ADC-33d	Pro213Arg; Ala218 dup (SANC 218a); Gly222Ser	
ADC-73	Gly222Ser	
ADC-162	Ala220Glu	
ADC-212	Ala200Asp; Ala218_Pro219 insLeu (SANC 218a); Pro219Ala	
ADC-212a	Ala200Asp; Ala218_Pro219 insLeu (SANC 218a); Pro219Ala; Gly212Asp	
ADC-214	Pro192Ala; Gly212Ser	
ADC-219	Ala218 dup (SANC 218a); Gly212Asp	
ADC-224	Pro213Arg; Ala218 dup (SANC 218a)	
ADC-228	Arg210Leu; Pro213Arg; Ala218 dup (SANC 218a)	
ADC-229	Gly212Asp	
ADC-232	Tyr199Phe; Pro213Arg; Ala218 dup (SANC 218a)	
ADC-233	Pro213Arg; Ala218 dup (SANC 218a); Ala220Ser	
**R2 loop**		
ADC-25b		Val298Glu
ADC-25c		Asn296Asp
ADC-30a		Ile291Ser
ADC-143		Met293Ile; Lys294 del
ADC-223		Asn296 del
ADC-226		Asn287Thr
**Both loops**		
ADC-33a	Pro213Arg; Ala218 dup (SANC 218a)	Ile291Ser
ADC-33c	Pro213Arg; Ala218 dup (SANC 218a)	Ser286Asn
ADC-162a	Ala220Glu	Val292Gly; Lys294Ile
ADC-214a	Pro192Ala; Gly212Ser	Val292Gly
ADC-225	Pro213Arg; Asp217Asn; Ala218 dup (SANC 218a)	Val292Gly
ADC-225a	Pro213Arg; Ala218 dup (SANC 218a)	Val292Gly
ADC-227	Pro213Arg; Ala218 dup (SANC 218a)	Val292Trp
ADC-230	Pro192Ala; Gly212Ser	Ser286Asn

Table S3. Data collection and structure refinement statistics.

Data set	Free form	avibactam complex		
		**50 mM, 4 h**	**50 mM, 24 h**	**100 mM, 4h**
**Data-collection**				
Source	Photon Factory BL-17A	Photon Factory BL-17A	Photon Factory BL-17A	Photon Factory BL-17A
wavelength (Å)	0.9800	0.9800	0.9800	0.9800
Space group	$P3_121$	$P3_121$	$P3_221$	$P3_221$
Unit-cell parameters				
length (Å)	$a = b = 76.0, c = 97.8$	$a = b = 73.8, c = 100.6$	$a = b = 75.7, c = 206.7$	$a = b = 76.1, c = 207.6$
Resolution range (Å)	48.92–1.95 (2.06–1.95)	39.52–1.90 (2.01–1.90)	47.49–2.10 (2.23–2.10)	47.7–2.11 (2.23–2.11)
No. of observed reflections	470,530 (75,992)	497,316 (79,419)	795,066 (128,386)	807,627 (127,056)
No. of unique reflections	24,405 (3,793)	25,649 (4,016)	40,905 (6,433)	41,103 (6,466)
Multiplicity	19.3 (20.0)	19.4 (19.8)	19.4 (19.6)	19.6 (19.6)
Completeness (%)	99.6 (97.6)	99.8 (98.9)	99.8 (98.8)	99.7 (98.5)
R_{merge}(%)[a]	9.0 (98.9)	9.3 (137.0)	9.1 (216.7)	6.6 (118.7)
$CC_{1/2}$	99.9 (86.7)	99.9 (82.8)	99.9 (70.7)	100 (83.7)
$\langle I/\sigma(I)\rangle$	18.70 (2.21)	16.30 (1.70)	17.43 (1.59)	23.50 (2.15)
**Refinement**				
Resolution (Å)	39.3–1.95 (2.02–1.95)	39.5–1.90 (1.97–1.90)	40.6–2.10 (2.15–2.10)	47.7–2.11 (2.16–2.11)
Reflection used	24,400 (2,564)	25,641 (2,728)	40,884 (2,598)	41,089 (2,588)
R_{work} (%)[b]	20.2 (29.8)	21.0 (30.6)	25.5 (37.2)	25.1 (36.9)
R_{free} (%)[c]	21.4 (35.5)	23.4 (33.0)	29.6 (41.2)	28.8 (42.3)
No. of non-hydrogen atoms	3,035	2,941	5,625	5,670
Protein	2,862	2,817	5,540	5,576
Ligands	15	17	34	34
Solvent	158	107	51	60
r.m.s.d. from ideality				
bond length (Å)	0.002	0.002	0.002	0.001
bond angle (°)	0.485	0.511	0.458	0.439
Average *B*-factor	47.7	54.0	79.3	77.4
Protein	47.7	54.1	79.5	77.5
Ligands	70.3	54.1	74.7	75.9
Solvent	46.7	49.7	62.6	62.7
Ramachandran plot				
favored region (%)	96.66	96.93	95.38	94.82
allowed region (%)	3.34	2.79	4.62	5.04
outlier region (%)	0.00	0.28	0.00	0.14
Clashscore	3.47	4.29	5.48	4.44
PDB ID	9WIP	9WIQ	9WIR	9WIS

Values in parentheses denote the highest resolution shell.
[a] $R_{\mathrm{merge}} = 100 \times \Sigma_{hkl} \Sigma_i | I_i(hkl) - \langle I(hkl)\rangle | / \Sigma_{hkl} \Sigma_i I_i(hkl)$, where $\langle I(hkl)\rangle$ is the mean value of $I(hkl)$.
[b] $R_{\mathrm{work}} = 100 \times \Sigma_{hkl} | |F_o| - |F_c| | / \Sigma_{hkl} |F_o|$, where F_o and F_c the observed and calculated structure factors, respectively. [c] R_{free} is calculated as for R_{work}, but for the test set comprising 5% reflections not used in refinement.

Table S4. Primers used for chromosomal recombineering of *A. baylyi*.

**PCR 1 (~500 bp upstream of *adc* gene)**	
**Primer name**	**Sequence (5'–3')**
All_ADCs _upstream_FOR	TGAGCGCCTTGCCTGATG
ADC (30_33)_upstream_REV	ACAAGAAATTTTTTTAAATCGCATTTAAAATGATTAATCCG TTACTGTG
ADC (227)_upsteam_REV	CTTTTTATTATTTTGAATAACACCCACTTAAAATGATTAATC CGTTACTGTG

**PCR 2 (*adc* gene from clinical strains)**	
**Primer name**	**Sequence (5'–3')**
ADC (30_33)_gene_FOR	CACAGTAACGGATTAATCATTTTAAATGCGATTTAAAAAAA TTTCTTGT
ADC (30_33)_gene_REV	GAAAAATAAACAAATAGGGGTTCCGCGTTATTTCTTTATTG CATTCAG
ADC (227)_gene_FOR	CACAGTAACGGATTAATCATTTTAAGTGGGTGTTATTCAAA ATAATAAAAAG
ADC (227)_gene_REV	GAAAAATAAACAAATAGGGGTTCCGCGTTATTCCTTTATTG CATTTAAAAC

**PCR 3 (gentamicin cassette)**	
**Primer name**	**Sequence (5'–3')**
ADC (30_33)_GENT_FOR	CTGAATGCAATAAAGAAATTACGCGGAACCCCTATTTGTTT ATTTTTC
ADC (227)_GENT_FOR	GTTTTAAATGCAATAAAGGAATAACGCGGAACCCCTATTTG TTTATTTTTC
All_ADCs_GENT_REV	CAATTTGAATCTACAATCAAATAAATTAGGTGGCGGTACTT GGGTC

**PCR 4 (~500 bp downstream of *adc* gene)**	
**Primer name**	**Sequence (5'–3')**
All_ADCs_downstream_FOR	GACCCAAGTACCGCCACCTAATTTATTTGATTGTAGATTCA AATTG
All_ADCs_downstream_REV	CACCGCCTGAACCGAGCG

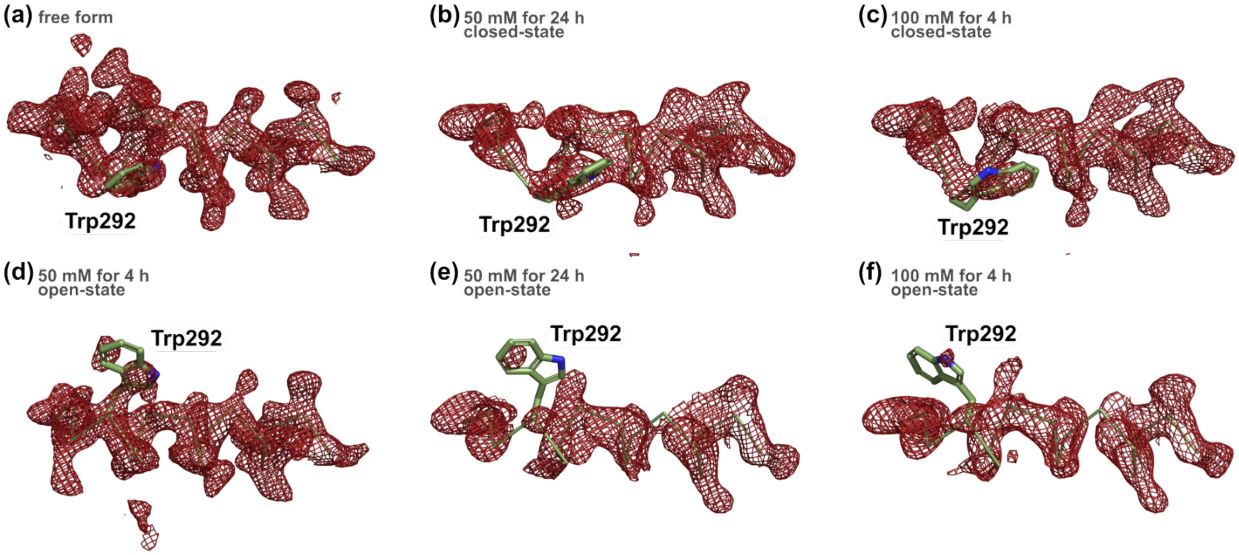

Figure S1. The R2 loop structures of ADC-227.

The R2 loop structure is shown as a ribbon representation, and the Trp292 residue is shown as a stick representation. The 2mF_o–DF_c map is shown as a red mesh contoured 1σ. (a) Free form. (b, e) The avibactam complex obtained under the condition of 50 mM for 24 h. (c, f) The avibactam complex obtained under the condition of 100 mM for 4 h. (d) The avibactam complex obtained under the condition of 50 mM for 4 h.
